# Pets in Voluntary Household Quarantine

**DOI:** 10.3201/eid1206.051548

**Published:** 2006-06

**Authors:** J. Scott Weese, Stephen A. Kruth

**Affiliations:** * University of Guelph, Guelph, Ontario, Canada

**Keywords:** SARS, pets, household quarantine, commentary

Outbreaks of severe acute respiratory syndrome (SARS) have resulted in increased discussion about community-based infection control measures, including voluntary quarantine. In the 2003 SARS outbreak in Toronto, Canada, at least 23,000 persons participated in voluntary quarantine in their homes because of possible exposure (*1*). Quarantined persons were told to remain at home, not allow anyone to visit, wear a mask when in the same room as other members of the household, and sleep in a separate room (*2*). These protocols were developed to decrease the risk of transmitting the SARS coronavirus to persons in the household. This situation highlights 1 aspect of community-based quarantine that has been overlooked: the potential role of household pets in disease transmission.

When SARS was first identified, potential host animal species were unknown, as was the risk of transmission between animals and humans. Despite the severity of SARS, the lack of information on the potential for interspecies transmission, and the potential implications of animals acting as reservoirs of infection, we are unaware of quarantine protocols that consider household pets. No specific data are available on pet ownership by quarantined persons; however, based on the prevalence of pet ownership in Canada, we assume that thousands of quarantined persons had household pets. Whether any precautions were taken to reduce the risk of SARS transmission to pets is unclear. Presumably, household pets had prolonged close contact with many quarantined persons. Additionally, many of these pets may have had close contact with other persons, both inside and outside the home, and contact with other animals. We now know that domestic cats and ferrets are susceptible to experimental infection by the SARS coronavirus and that they can transmit this virus to other cats and ferrets (*3*). What would have happened if cats were naturally infected in households and could transmit infection to humans or other animals? Were measures in place to reduce the risk for this transmission and detect it had it occurred? If SARS had established itself in the feral cat population in affected cities, would it have been controllable?

Although SARS is the most recent example of an emerging disease for which quarantine was implemented, the potential for household transmission through pets should be considered in any new disease when information is incomplete regarding potential hosts and the risk for interspecies transmission. If one considers that an estimated 75% of transmissible emerging diseases are zoonoses (*4*), the relevance becomes clear.

While most of the discussion of zoonoses has focused on food-producing animals and wildlife, companion animals require closer scrutiny because of the number of persons exposed to pets and the nature of human-animal interaction. Pets are present in ≈58.3% of households in the United States; the pet population includes ≈62 million dogs, ≈69 million cats, ≈10 million birds, and ≈3 million reptiles (*5*). Also included are smaller numbers of ferrets, rabbits, rats, hamsters, hedgehogs, and other small mammals and exotic species. Many, if not most, owners of household pets likely have more prolonged and close contact with their pets than with most other persons. Ample reports exist regarding transmission of bacterial, viral, and fungal pathogens between humans and pets (in both directions) in the household (*6**–**11*). In addition to SARS, some pathogens that have recently been identified as of concern include methicillin-resistant *Staphylococcus aureus* (*12*), monkeypox (*6*), and H5N1 influenza (*13*). Although transmission of pathogens from domestic pets often focuses on the household, many other persons also have regular or sporadic contact with household pets owned by friends or family or through animal visitation programs.

Development of community-based quarantine protocols that consider the role of domestic animals in transmission of disease remains a gap in current preparedness planning activities. We believe that the potential role of household pets should be considered in transmission of all emerging infectious diseases. This would include promptly and thoroughly evaluating the susceptibility of pets of various species to clinical disease and subclinical infection and assessing the possibility of transmission of pathogens between humans and pets, in both directions. Community-based quarantine measures may need to address contingency protocols for placing household pets in quarantine as well as human family members. Among the factors that need to be evaluated are the following: when pets should be quarantined, what type of unprotected animal-human contact should be allowed, what types of outdoor access by pets should be allowed (if any), what infection control measures should be implemented in the household to decrease the risk of pathogen transmission, how pet fecal material should be handled in the household and outdoors and in community settings, and what measures should be taken when and if veterinary care is required. Additionally, clinical and epidemiologic studies involving household pets may be indicated during the emergence of infectious diseases to evaluate the potential role of pets in disease transmission, to help manage disease in pets, and to determine whether pets may act as sentinel species.

We recommend that a coordinated effort between the human and veterinary medical fields and public health authorities be undertaken to address these issues. Relevant groups would involve national or regional regulatory bodies, public health agencies, infection control specialists in the human and veterinary fields, veterinary organizations, primary care veterinarians, laboratory animal veterinarians, comparative medicine specialists, and humane society personnel. Because of the number of groups that should be included and the potential complexity of the situation, proactive planning is needed.
